# A randomized phase II clinical trial of stereotactic body radiation therapy (SBRT) and systemic pembrolizumab with or without intratumoral avelumab/ipilimumab plus CD1c (BDCA-1)^+^/CD141 (BDCA-3)^+^ myeloid dendritic cells in solid tumors

**DOI:** 10.1007/s00262-024-03751-0

**Published:** 2024-07-02

**Authors:** Manon Vounckx, Jens Tijtgat, Latoya Stevens, Iris Dirven, Bart Ilsen, Frederik Vandenbroucke, Steven Raeymaeckers, Karolien Vekens, Ramses Forsyth, Xenia Geeraerts, Ivan Van Riet, Julia Katharina Schwarze, Sandra Tuyaerts, Lore Decoster, Mark De Ridder, Ines Dufait, Bart Neyns

**Affiliations:** 1 Department of Medical Oncology, Laboratory for Medical and Molecular Oncology (LMMO), Vrije Universiteit Brussel (VUB), Universitair Ziekenhuis Brussel (UZ Brussel), Laarbeeklaan 101, 1090 Brussels, Belgium; 2 Department of Radiology, Universitair Ziekenhuis Brussel (UZ Brussel), Laarbeeklaan 101, 1090 Brussels, Belgium; 3 Department of Pathology, Vrije Universiteit Brussel (VUB), Universitair Ziekenhuis Brussel (UZ Brussel), Laarbeeklaan 101, 1090 Brussels, Belgium; 4 Department of Radiotherapy, Universitair Ziekenhuis Brussel (UZ Brussel), Laarbeeklaan 101, 1090 Brussels, Belgium; 5 Department of Hematology, Stem Cell Laboratory, Vrije Universiteit Brussel (VUB), Universitair Ziekenhuis Brussel (UZ Brussel), Laarbeeklaan 101, 1090 Brussels, Belgium

**Keywords:** Myeloid dendritic cell therapy, Intratumoral immunotherapy, Stereotactic body radiation therapy, Melanoma, Non-small lung cell carcinoma

## Abstract

**Background:**

Radiotherapy (RT) synergizes with immune checkpoint blockade (ICB). CD1c(BDCA-1)^+^/CD141(BDCA-3)^+^ myeloid dendritic cells (myDC) in the tumor microenvironment are indispensable at initiating effector T-cell responses and response to ICB.

**Methods:**

In this phase II clinical trial, anti-PD-1 ICB pretreated oligometastatic patients (tumor agnostic) underwent a leukapheresis followed by isolation of CD1c(BDCA-1)^+^/CD141(BDCA-3)^+^ myDC. Following hypofractionated stereotactic body RT (3 × 8 Gy), patients were randomized (3:1). Respectively, in arm A (immediate treatment), intratumoral (IT) ipilimumab (10 mg) and avelumab (40 mg) combined with intravenous (IV) pembrolizumab (200 mg) were administered followed by IT injection of myDC; subsequently, IV pembrolizumab and IT ipilimumab/avelumab were continued (q3W). In arm B (contemporary control arm), patients received IV pembrolizumab, with possibility to cross-over at progression. Primary endpoint was 1-year progression-free survival rate (PFS). Secondary endpoints were safety, feasibility, objective response rate, PFS, and overall survival (OS).

**Results:**

Thirteen patients (10 in arm A, eight non-small cell lung cancer, and five melanoma) were enrolled. Two patients crossed over. One-year PFS rate was 10% in arm A and 0% in arm B. Two patients in arm A obtained a partial response, and one patient obtained a stable disease as best response. In arm B, one patient obtained a SD. Median PFS and OS were 21.8 weeks (arm A) versus 24.9 (arm B), and 62.7 versus 57.9 weeks, respectively. An iatrogenic pneumothorax was the only grade 3 treatment-related adverse event.

**Conclusion:**

SBRT and pembrolizumab with or without IT avelumab/ipilimumab and IT myDC in oligometastatic patients are safe and feasible with a clinically meaningful tumor response rate. However, the study failed to reach its primary endpoint.

*Trial registration number*: Clinicaltrials.gov: NCT04571632 (09 AUG 2020). EUDRACT: 2019-003668-32.* Date of registration*: 17 DEC 2019, amendment 1: 6 MAR 2021, amendment 2: 4 FEB 2022.

**Supplementary Information:**

The online version contains supplementary material available at 10.1007/s00262-024-03751-0.

## Introduction

Immune checkpoint blockade (ICB) has significantly improved survival in melanoma and non-small-cell lung cancer (NSCLC); yet, response rates remain low [1, 2]. Managing oligoprogression, i.e., the emergence of a few lesions post-initial systemic therapy success, is challenging [3].

The cancer-immunity cycle underscores dendritic cells' (DC) pivotal role in priming CD4^+^ and CD8^+^ T-cells through presentation of tumor-associated antigens (TAAs) in tumor-draining lymph nodes (TDLN) [4]. In the tumor microenvironment (TME), DC plays a crucial role, divided into plasmacytoid DC (pDC) and conventional (cDC) or myeloid DC (myDC), further categorized as CD141 (BDCA-3)^+^ or cDC1 and CD1c (BDCA-1)^+^ or cDC2 [5]. While cDC1 primarily drives CD8^+^ cytotoxic T-lymphocytes (CTL) responses and cDC2 activates CD4^+^ T-lymphocytes within the TME, inadequate DC recruitment under tumor growth conditions hampers anti-tumor immunity [4, 6]. Excluding cDC from the TME diminishes ICB efficacy, resulting in defective CTL activation and facilitating metastases evasion [7]. Furthermore, an immunosuppressive phenotype marked by PD-L1 expression (referred to as cDC3, LAMP3 + DCs, or mregDCs) has been identified [5]. Limited (pre-)clinical trials have explored the isolation and intratumoral/intranodal administration of myDC and myDC-derived cellular vaccines [8].

Stereotactic body radiation therapy (SBRT) has emerged as a treatment for oligoprogression, particularly in NSCLC patients [9]. Preclinical studies indicate that SBRT induces a decrease in CD8^+^ T-cells and cDC1 and an increase in cDC2 and CD4^+^/Treg T-cell ratio supporting intratumoral immune cell repertoire boosting, with little understanding of optimal dose and fractionation [10]. Combining low-dose-fractionated radiotherapy (3 × 8 Gy) with anti-CTLA-4 antibodies has shown promise in inducing abscopal effects through cDC1 activation in preclinical models. Despite SBRT's local immunosuppressive effect, it can trigger a systemic anti-tumor immune response, enhancing both priming and effector stages [11]. SBRT induces immunogenic cell death (ICD), releasing TAAs that activate DC, especially the cDC1 subset, which migrate to TDLN to prime CD8^+^ T-cells [12]. SBRT-induced cell death activates toll-like receptor 4 and type 1 interferon signaling and boosts antigen processing and presentation by increasing major histocompatibility complex class I levels, suggesting the potential of combining DC treatment with SBRT as a strategy to enhance anti-tumor immune response [13, 14].

This study combines the cDC presence in the TME with the immunogenic effect of SBRT through ICD induction. We investigated the efficacy and toxicity of SBRT (3 × 8 Gy) combined with an intratumoral (IT) injection of CD1c(BDCA-1)^+^/CD141(BDCA-3)^+^ myDC, anti-PD-L1, and anti-CTLA-4. Previously, we reported the combination of myDC and IT ICB to be safe [15]. We aimed to assess whether this combination enhances responses in advanced solid tumor patients with oligoprogression on anti-PD-1 therapy compared to SBRT alone.

## Methods

### Patients

Patients with unresectable advanced solid tumors, presenting with oligoprogressive disease (defined as ≤ 7 sites) on anti-PD1 therapy (pembrolizumab or nivolumab) alone or in combination (anti-CTLA-4 ICB or chemotherapy), were eligible. Initially limited to NSCLC, the trial expanded to all solid tumors in January 2022 due to slow recruitment. At least one lesion (> 1 cm) needed to be injectable clinically or imaging-guided and be eligible for SBRT. Other criteria included age ≥ 18, ECOGps < 2, normal hematological/liver/renal function, and negative HIV/syphilis/hepatitisserology. Exclusions comprised untreated central nervous system metastases, autoimmune disease, immunosuppressive treatment, grade 3–4 immune-related adverse events (AEs), malignancy in the past 5 years, and contraindication to stop anticoagulation.

### Study design and treatment

This phase II randomized trial investigated the combination of SBRT and pembrolizumab with or without intratumoral avelumab/ipilimumab plus autologous CD1c (BDCA-1)^+^/CD141 (BDCA-3)^+^ myDCs (Supplementary Fig. S1). After screening, patients underwent leukapheresis. Following SBRT to progressive lesions (3 × 8 Gy), patients were randomized (3:1) to an immediate treatment arm (A) or a contemporary control arm (B). In arm A, 10 mg of ipilimumab (Yervoy®) and 40 mg of avelumab (Bavencio®) were injected in the chosen lesion along with intravenous (IV) administration of 200 mg pembrolizumab (KeytrudaTM). In the following day, autologous myDCs (suspension volume based on lesion size) were injected in the same lesion. Patients continued IV pembrolizumab along with IT ipilimumab and avelumab Q3W. In arm B, patients received IV pembrolizumab 200 mg Q3W, with possibility to start IT injections upon progressive disease (PD). Approval was obtained of Universitair Ziekenhuis Brussel ethics committee (2019-440) and the Belgian Federal Agency for Medicines and Health Products (EudraCT2019-003668-32).

### Leukapheresis, isolation, and cryopreservation of myeloid dendritic cells

Leukapheresis and myDC isolation were previously described in J. Tijtgat et al., 2024. The cryopreservation procedure is described in supplementary methods.

### Assessment of response and toxicity

Tumor responses were evaluated using whole-body 18-fluorodeoxyglucose positron emission tomography-computed tomography (^18F^FDG-PET/CT) every 12 weeks, with objective response rates (ORR) defined by Response Evaluation Criteria in Solid Tumors for Immunotherapy (iRECISTv1.1). Clinical examinations and blood analyses were conducted before each treatment cycle, and AEs were documented using CTCAE (v5.0) by type, frequency, and severity.

### Determination of lymphocyte subsets and HMGB1 in peripheral blood

Peripheral blood was collected from all patients at baseline (= during leukapheresis), at D-5 (start of SBRT), D1 (start of IT and IV treatment) and every Q3W thereafter. The determination of lymphocyte subsets and HMGB1 is described in the supplementary methods.

### Tumor biopsies and immunohistochemistry

When safe and feasible, core-needle tumor biopsies were collected on D1 and every Q3W. Immunohistochemistry (IHC) stainings for CD3 (clone 2GV6) and CD8 (clone SP57) were routinely performed while SOX10 (clone SP267), PD-L1 (clone 22C3, SP263), and, in case of lung tissue, CK 7 (clone SP52), were stained occasionally (all clones Roche®).

### Statistical analysis

The study's primary endpoint is 1-year progression-free survival (1YPFS) rate following randomization. Based on a historical 1YPFS rate of 45% (as reported by Collen et al., 2014) in a cohort of 26 oligometastatic (≤ 5) NSCLC patients treated with SBRT after induction chemotherapy (*n* = 17) or as primary treatment (*n* = 9), the desired 1YPFS rate for arm A was set at 65% [16]. Using a Fleming one-stage design (null hypothesis p_0_ 45%, alternative hypothesis p_1_ 65%,* α* = 0.1 and *β* = 0.2), the required sample size of arm A was 27 patients and nine in arm B. Safety, feasibility, ORR by iRECIST, response of (non-)injected tumor sites, duration of response, PFS, and overall survival (OS) were secondary endpoints.

Descriptive statistics were used for baseline characteristics, treatment administration, efficacy endpoints, and AE. Demographics and study drug administration were tabulated, including cycle count, dose intensity, modifications, and reason for deviations. 1YPFS rate and OS were determined using Kaplan–Meier survival estimates. Simple linear regression analysis was applied to study correlations. Data were analyzed using GraphPad Prism v10.

Database was locked on November 24, 2023

### Results

#### Patient characteristics

**Fig. 1:**
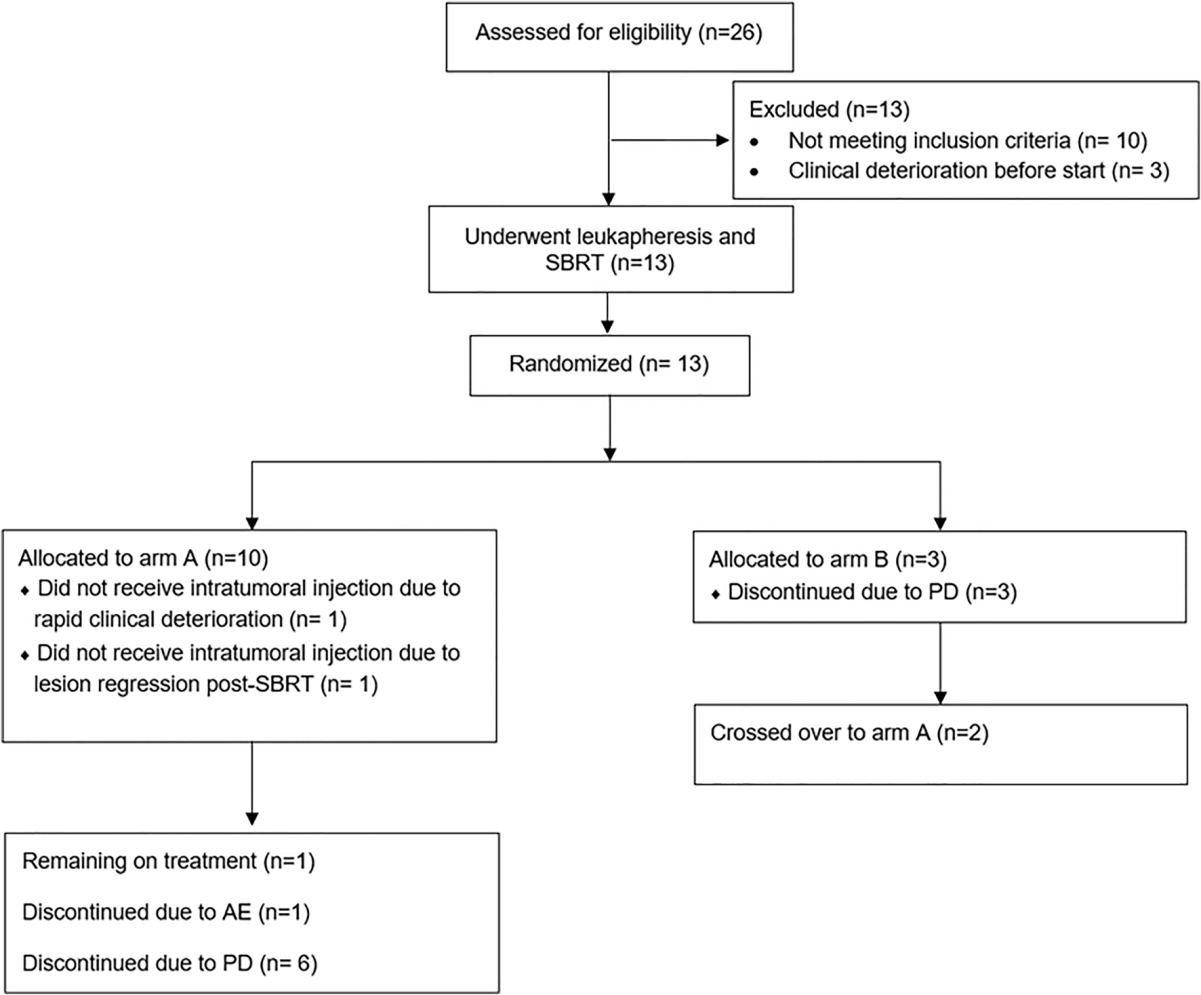
CONSORT diagram: 13 patients were recruited between September 2020 and June 2023

Thirteen patients (nine male and four female) were recruited (CONSORT diagram in Fig. 1). The trial was terminated prematurely because of slow recruitment, partly related to the COVID-19 pandemic. Additionally, patient inclusion was hindered by advanced disease stages, rapid disease progression, and prior radiation therapy to target lesions.

Ten patients were randomized to arm A and three to arm B. The baseline characteristics per treatment arm are in Table 1. Individual patient tumor types and stages are reported in Supplementary Table S1.

**Table 1:** Baseline characteristics of included patients

Patient characteristics		
	Arm A (*n* = 10)	Arm B (*n* = 3)
Median age, years (range)	61.5 (35–73)	55 (35–65)
Sex, n (*%*)		
Male	7 (*70*)	2 (*66.6*)
Female	3 (*30*)	1 (*33.3*)
ECOG performance status, n (*%*)		
0	7 (*70*)	3 (*100*)
1	3 (*30*)	
Tumor type and stage, n (%)		
NSCLC	6 (*60*)	2 (*66.6*)
Stage IV adenocarcinoma	6	2
Cutaneous melanoma	3 (*30*)	1 (*33.3*)
Stage IV-M1a	0	1
Stage IV-M1b	2	0
Stage IV-M1c	1	0
Uveal melanoma (stage IV)	1 (10*%*)	0
Prior systemic therapy (%)		
Anti-PD-1	10 (*100*)	3 (*100*)
Anti-CTLA-4	1	1
Chemotherapy	5	1
Prior lines of therapy, median (range)	2 (1–4)	3 (1–3)

### Treatment disposition

Patients received a median of 4 IT (arm A) and 7 IV administrations. One patient in arm A discontinued IT injections, while two patients could not receive any due to clinical deterioration or lesion shrinkage post-radiation. Additionally, two patients crossed over to arm A at progression (median of 3.5 IT and 15 IV administrations).

### Isolation and characterization of myDC

All patients were able to undergo leukapheresis, and no unexpected AEs were observed. Isolation of CD1c (BDCA-1)^+^ and CD141 (BDCA-3)^+^ myDC was successful in all but one patient due to a technical issue. Median numbers and phenotypes of myDC (CD1c/CD141) are summarized in Supplementary Table S1.

### Safety and adverse events (AEs)

Most common AEs were fatigue (38%), nausea (38%), and pneumonitis (30%). Three NSCLC patients developed grade 3 pneumonitis, requiring therapy discontinuation. Other discontinuation reasons included cerebrovascular ischemia (*n* = 1) and hypophysitis (*n* = 1). IT injections were generally well-tolerated. Grades 2 and 3 AEs are detailed in Table 2 (full list in Supplementary Table S2).

**Table 2:** Adverse events with grade ≥ 2; *n* (%); bold means most frequent grade 2 and 3 adverse events

Adverse events grade 2 and 3				
	CTCAE v 5.0 grade			
	Grade 2		Grade 3	
	Arm A	Arm B	Arm A	Arm B
Adrenal insufficiency			1 (10)	
Amenorrhea		1 (33.3)	1 (10)	
Constipation		1 (33.3)	1 (10)	
**Fatigue**	**2** (20)		**1** (10)	
Hypertension			1 (10)	
Hypophysitis			1 (10)	
Hypothyroïdism		1 (33.3)		
Injection site reaction	**2** (20)			
Ischemia cerebrovascular			1 (10)	
Malaise	1 (10)			
**Nausea**	**3** (30)	**1** (33.3)		
Peripheral sensory neuropathy	1 (10)			
**Pneumonitis**	**2** (20)	**1** (33.3)		
Pneumothorax				1 (33.3)
Skin infection	1 (10)			
Tumor Pain	1 (10)			
Vomiting	1 (10)			
Weight loss	1(10)			
**Total**	**15**	**5**	**5**	**1**

### Efficacy/clinical outcome

**Fig. 2:**
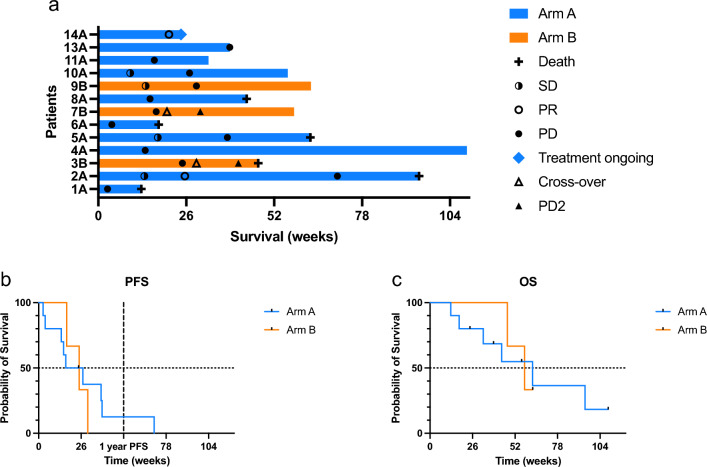
Swimmer plot (**a**) of randomized patients, Kaplan–Meier curve of progression-free survival (PFS) (**b**), and overall survival (OS) (**c**). *Abbreviations: stable disease (SD), partial response (PR), progressive disease (PD), progressive disease after cross-over (PD2)*

**Fig. 3:**
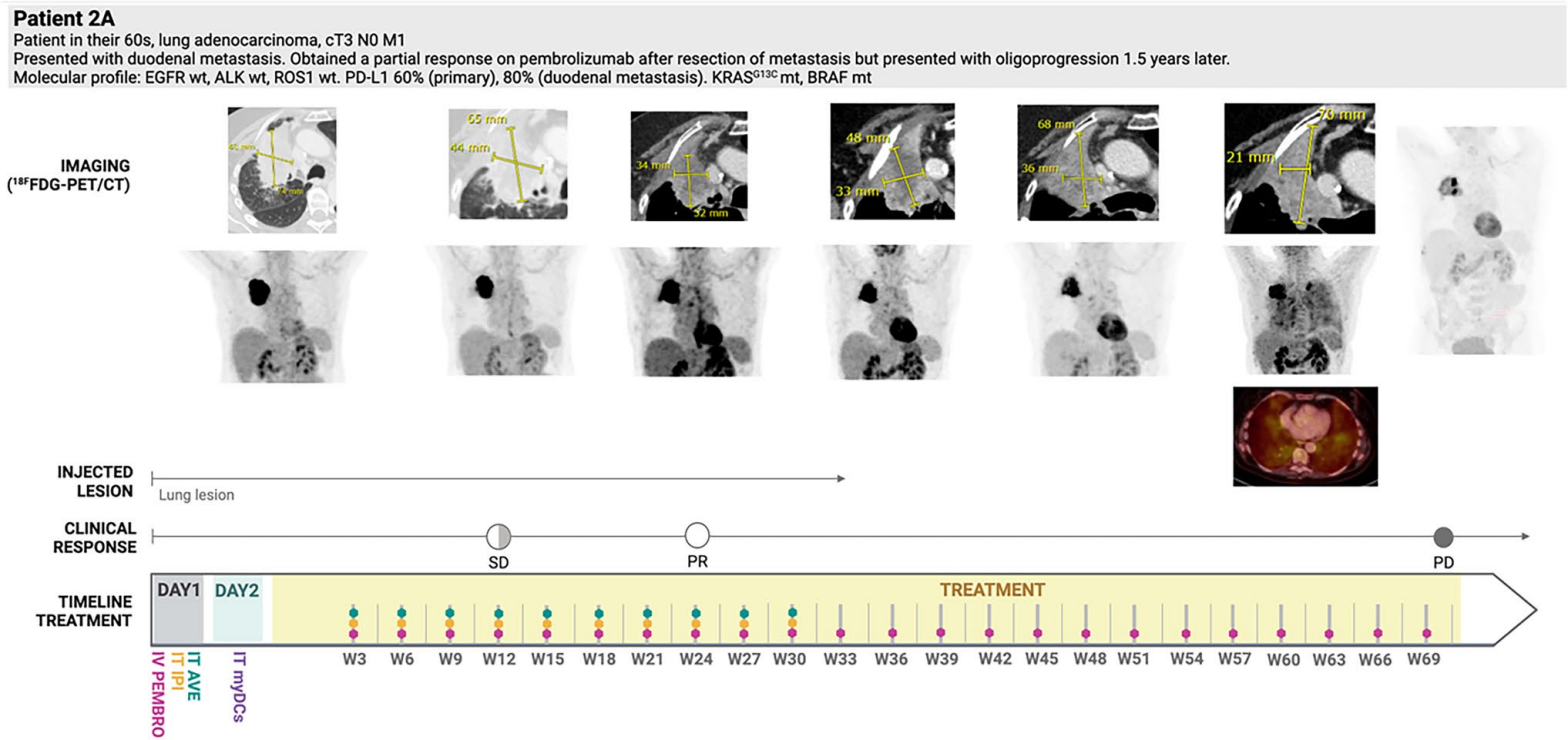
Case illustration of patient 2 (arm A), who had a PR at week 25, but encountered IT treatment interruptions (at week 33) due to pain and fibrosis. Hospitalization for fever, elevated inflammatory markers, and widespread lung activity on PET-CT was managed by high-dose corticosteroids. A relapse of the latter followed by a COVID-19 infection led to further treatment interruption with disease progression by week 70. Upper row images: CT, second row: PET/CT maximum intensity projections (MIP) of disease locations, third row (single image): fused PET/CT image illustrating diffuse PET activity in both lungs, suggestive of (ir-)pneumonitis. *SD* = *stable disease, PR* = *partial response, PD* = *progressive disease, PEMBRO* = *pembrolizumab, AVE* = *avelumab, IPI* = *ipilimumab*

All patients (*n* = 13) were evaluable for tumor response. In this mixed population of melanoma (*n* = 4) and NSCLC (*n* = 8) patients, we observed two partial responses (PR) (patient 2A NSCLC and 14A melanoma, ongoing for 15.6 and 6 months, respectively), yielding an ORR of 20% in arm A. No objective responses were observed in arm B. Stable disease (SD) was objectified in two patients of arm A (patient 5A NSCLC and 10A melanoma) and one patient in arm B (patient 9B melanoma) (Fig. 2a). The 1YPFS rate was 10% in arm A and 0% in arm B. Median PFS and OS were 21.8 and 62.7 weeks in arm A, respectively, and 24.9 and 57.9 weeks in arm B (Fig. 2b, c). Two of three patients in arm B progressed and crossed over to IT injections, with a median PFS of 11.14 weeks thereafter. A case illustration is shown in Fig. 3.

### Effect of treatment on blood immune cell composition and immunohistochemistry

At every IT injection, five lymphocyte subsets of PBMCs were analyzed. Two patients (1A, 6A) exhibited PD shortly after treatment initiation (Fig. 2), halting further longitudinal sample collection. Baseline naive T-cell levels (defined as CD45RA^+^CD45RO^−^CD27^+^CCR7^+^CD62L^+^) were low, likely due to prior treatments. No significant changes were observed in naive, effector (CD45RA^+^CD45RO^−^CD27^−^CCR7^−^CD62L^−^), effector memory (CD45RA^−^CD45RO^+^CD27^+^CCR7^+^CD62L^+^), and central memory T-cells (CD45RA^−^CD45RO^+^CD27^+^CCR7^+^CD62L^+^) between arm A and B due to high interpatient variability. Additionally, no significant changes were observed when comparing T-cell subsets at baseline (D-5) and post-treatment (D1 and Q3W thereafter) (data not shown). A variable increase in Tregs was observed directly post-SBRT (D1) as compared to pre-SBRT (D-5), with a subsequent normalization of Treg levels (Supplementary Fig. S2).

Supplementary figures 3a and 3b provide an overview of immune checkpoint expression on CD8^+^ and CD4^+^ T-cells, respectively.

We hypothesized that adding SBRT induces ICD, enhancing anti-tumoral immune responses. Plasma HMGB1 levels, an ICD marker, remained stable in eight patients but significantly increased post-SBRT in four patients (5A, 10A (both SD), 11A, and 13A (both PD)) (Supplementary Fig. S4). No correlation was found between irradiated volumes and HMGB1 increase (Supplementary Fig. S5). Surprisingly, Patient 1A showed decreased HMGB1 levels post-SBRT despite initial high levels. Some patients showed increased PD-L1 and CD8^+^ T-cell levels in tumor biopsies (Supplementary Fig. S6).

## Discussion

This randomized phase II clinical trial investigated the combination of SBRT, IV and IT ICB, and IT administration of myDC in anti-PD-1 pretreated patients. Despite demonstrating safety and feasibility, the observed anti-tumor activity in the experimental arm did not meet expectations, leading to recruitment termination. However, clinically meaningful activity was noted in a minority of patients. A challenge was identifying eligible candidates, with some excluded due to early clinical deterioration, and others having undergone prior radiation therapy, particularly in the NSCLC population, making the intended radiation dose unsafe and unfeasible.

While leukapheresis was successful in all but one patient, IT administration feasibility was suboptimal. Limited efficacy was seen in three patients in arm A, with a shorter PFS compared to the historical cohort [16]. High pneumonitis incidence, by combining ICB and radiotherapy, particularly in NSCLC patients, was noted, hindering tumor biopsy collection [17].

Combining ICB and SBRT aims to reduce immunosuppression and induce ICD in the TME, enhancing the anti-tumor immune response. However, prospective trials have not consistently shown significant efficacy improvements [18]. Implementing stricter selection criteria, like excluding patients with prior radiation at disease progression sites and ensuring optimal performance scores before treatment, may enhance response rates.

Determining the best timing and dosage of SBRT alongside ICB remains elusive, as shown in the Belgian CHEERS trial, where SBRT (3 × 8 Gy) addition did not improve PFS or OS [19]. Optimal timing of ICB with radiation varies based on the immunotherapy mechanism, while conflicting data on ideal radiation dosage for synergy with ICB underscores the need for preclinical exploration [14, 20]. Moreover, despite reported instances of an abscopal effect, its consistency and interaction with ICB merit further investigation, as our study did not observe such outcomes.

## Conclusion

Combining SBRT, ICB, and IT myDC proved safe and feasible in select patients, with promising activity. However, the primary 1YPFS endpoint was not met, leading to early study termination. Further investigation into SBRT timing and dosage, along with patient selection, is crucial to enhance the therapeutic effectiveness of this approach.

### Supplementary Information

Below is the link to the electronic supplementary material.

Supplementary file1 (PDF 1232 kb)

Supplementary file2 (DOCX 20 kb)

Supplementary file3 (PDF 873 kb)

## Abbreviations

1YPFS: 1-year progression-free survival rate
AEs: Adverse events
CD: Cluster of differentiation
cDC: Conventional or circulating dendritic cell(s)
CTL: Cytotoxic T-lymphocytes
DC: Dendritic cell(s)
ECOGps: Eastern cooperative oncology group performance status
ICB: Immune checkpoint blockade
iRECIST: Immunotherapy response evaluation criteria in solid tumors
IT: Intratumoral
IV: Intravenous
myDC: Myeloid dendritic cell(s)
NSCLC: Non-small-cell lung carcinoma
ORR: Objective response rate
OS: Overall survival
PBMC: Peripheral blood mononuclear cells
PD: Progressive disease
pDC: Plasmacytoid dendritic cell(s)
PFS: Progression-free survival
RT: Radiotherapy
SBRT: Stereotactic body radiation therapy
SD: Stable disease
TAAs: Tumor-associated antigens
TDLN: Tumor-draining lymph nodes
TME: Tumor microenvironment

## References

[1] Larkin J et al (2015) Combined nivolumab and ipilimumab or monotherapy in untreated melanoma. N Engl J Med 373(1):23–34 26027431 10.1056/NEJMoa1504030 PMC5698905

[2] Gandhi L et al (2018) Pembrolizumab plus chemotherapy in metastatic non-small-cell lung cancer. N Engl J Med 378(22):2078–2092 29658856 10.1056/NEJMoa1801005

[3] Guckenberger M et al (2020) Characterisation and classification of oligometastatic disease: a European Society for Radiotherapy and Oncology and European Organisation for Research and Treatment of Cancer consensus recommendation. Lancet Oncol 21(1):e18–e28 31908301 10.1016/S1470-2045(19)30718-1

[4] Broz ML et al (2014) Dissecting the tumor myeloid compartment reveals rare activating antigen-presenting cells critical for T cell immunity. Cancer Cell 26(6):938 28898680 10.1016/j.ccell.2014.11.010

[5] Pittet MJ et al (2023) Dendritic cells as shepherds of T cell immunity in cancer. Immunity 56(10):2218–2230 37708889 10.1016/j.immuni.2023.08.014 PMC10591862

[6] Gardner A, de Mingo Pulido A, Ruffell B (2020) Dendritic cells and their role in immunotherapy. Front Immunol 11:924 32508825 10.3389/fimmu.2020.00924 PMC7253577

[7] Salmon H et al (2016) Expansion and activation of CD103(+) dendritic cell progenitors at the tumor site enhances tumor responses to therapeutic PD-L1 and BRAF inhibition. Immunity 44(4):924–938 27096321 10.1016/j.immuni.2016.03.012 PMC4980762

[8] Schwarze JK et al (2023) Current “state of the art” on dendritic cell-based cancer vaccines in melanoma. Curr Opin Oncol 35(2):87–93 36721893 10.1097/CCO.0000000000000926

[9] Kagawa Y et al (2020) Efficacy of local therapy for oligoprogressive disease after programmed cell death 1 blockade in advanced non-small cell lung cancer. Cancer Sci 111(12):4442–4452 32770608 10.1111/cas.14605 PMC7734009

[10] Reijmen E et al (2021) Fractionated radiation severely reduces the number of CD8+ T cells and mature antigen presenting cells within lung tumors. Int J Radiat Oncol Biol Phys 111(1):272–283 33865948 10.1016/j.ijrobp.2021.04.009

[11] Dewan MZ et al (2009) Fractionated but not single-dose radiotherapy induces an immune-mediated abscopal effect when combined with anti-CTLA-4 antibody. Clin Cancer Res 15(17):5379–5388 19706802 10.1158/1078-0432.CCR-09-0265 PMC2746048

[12] Di Blasio S et al (2016) Human CD1c(+) DCs are critical cellular mediators of immune responses induced by immunogenic cell death. Oncoimmunology 5(8):e1192739 27622063 10.1080/2162402X.2016.1192739 PMC5007971

[13] Janopaul-Naylor JR, et al (2021) The Abscopal effect: a review of pre-clinical and clinical advances. Int J Mol Sci 22(20) 10.3390/ijms222011061 PMC8537037 34681719

[14] Dagoglu N et al (2019) Abscopal effect of radiotherapy in the immunotherapy era: systematic review of reported cases. Cureus 11(2):e4103 31057997 10.7759/cureus.4103 PMC6476623

[15] Schwarze JK, et al (2020) Intratumoral combinatorial administration of CD1c (BDCA-1)(+) myeloid dendritic cells plus ipilimumab and avelumab in combination with intravenous low-dose nivolumab in patients with advanced solid tumors: a phase IB clinical trial. Vaccines (Basel) 8(4) 10.3390/vaccines8040670 PMC7712037 33182610

[16] Collen C et al (2014) Phase II study of stereotactic body radiotherapy to primary tumor and metastatic locations in oligometastatic nonsmall-cell lung cancer patients. Ann Oncol 25(10):1954–1959 25114022 10.1093/annonc/mdu370

[17] Tyczynski JE et al (2021) Incidence and risk factors of pneumonitis in patients with non-small cell lung cancer: an observational analysis of real-world data. Oncol Ther 9(2):471–488 33909273 10.1007/s40487-021-00150-8 PMC8593090

[18] Turchan WT, Chmura SJ (2021) The role of immunotherapy in combination with oligometastasis-directed therapy: a narrative review. Ann Palliat Med 10(5):6028–6044 33440982 10.21037/apm-20-1528

[19] Spaas M, et al (2023) Checkpoint inhibitors in combination with stereotactic body radiotherapy in patients with advanced solid tumors: the CHEERS Phase 2 randomized clinical trial. JAMA Oncol 10.1001/jamaoncol.2023.2132 PMC10326732 37410476

[20] Young KH et al (2016) Optimizing timing of immunotherapy improves control of tumors by hypofractionated radiation therapy. PLoS ONE 11(6):e0157164 27281029 10.1371/journal.pone.0157164 PMC4900555

