# Supplementary material for: A randomized phase II clinical trial of stereotactic body radiation therapy (SBRT) and systemic pembrolizumab with or without intratumoral avelumab/ipilimumab plus CD1c (BDCA-1)+/CD141 (BDCA-3)+ myeloid dendritic cells in solid tumors
Source: Cancer Immunol Immunother. 2024 Jul 2;73(9):167. doi: 10.1007/s00262-024-03751-0 (PMC11219623; doi:10.1007/s00262-024-03751-0)
Supplement: Supplementary file 1 — Supplementary file1 (PDF 1232 kb) [file 262_2024_3751_MOESM1_ESM.pdf]

Supplementary figures

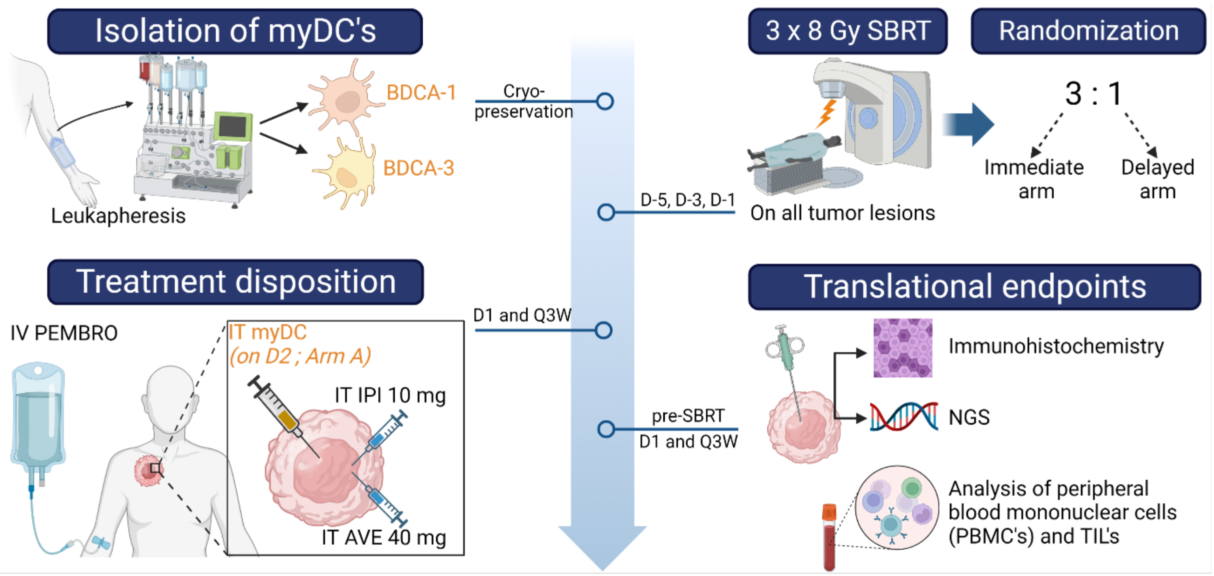

Supplementary figure 1: Clinical Trial design. *Abbreviations: SBRT: stereotactic body radiation therapy, PEMBRO: pembrolizumab, IPI: ipilimumab, AVE: avelumab, NGS: next generation sequencing, PBMCs: peripheral blood mononuclear cells, TIL: tumor infiltrating lymphocytes.*

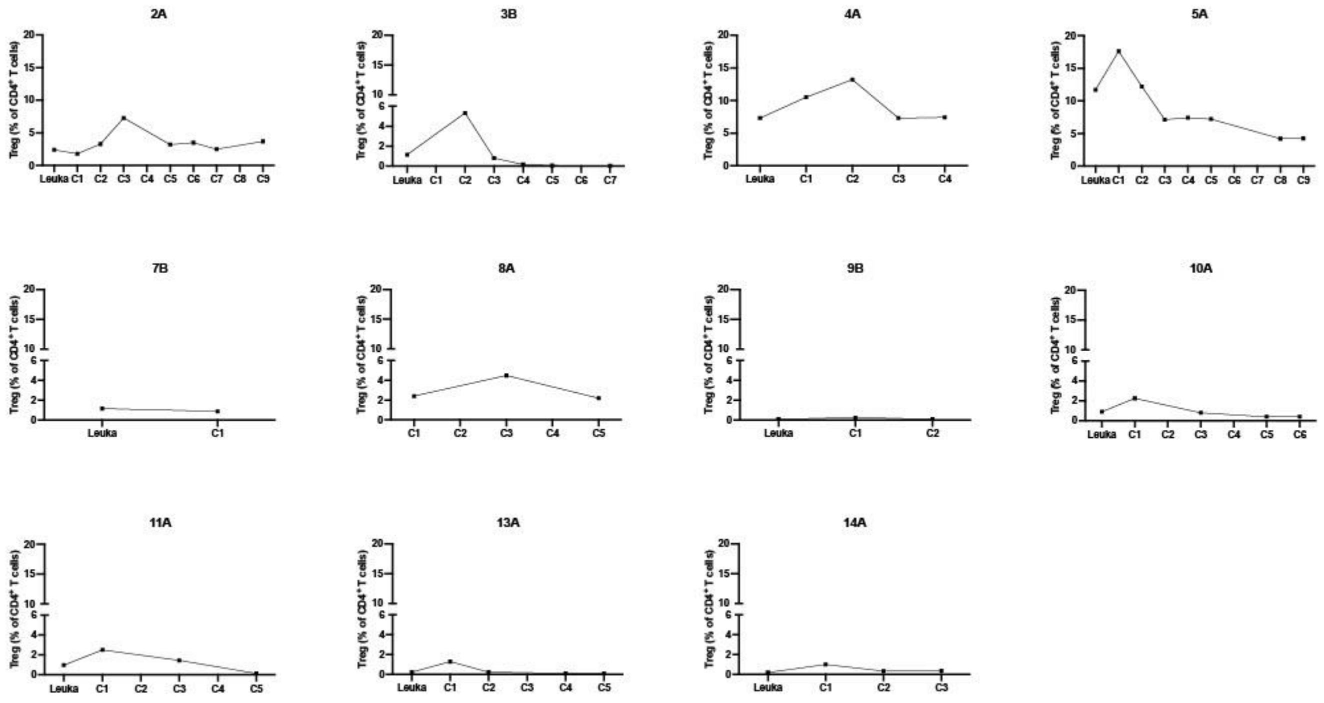

Supplementary figure 2: longitudinal analysis of Tregs (% of $CD4^+$ T cells) during the course of treatment, defined as $CD4^+CD25^+CD127^-Foxp3^+$ cells. Ki67 expression within circulating Tregs did not show any signs of recently activated/proliferating Tregs over the different time points. Leuka represents blood analysis at baseline. Blood analysis for every treatment cycle (Q3W) is depicted as C1, C2 etc.

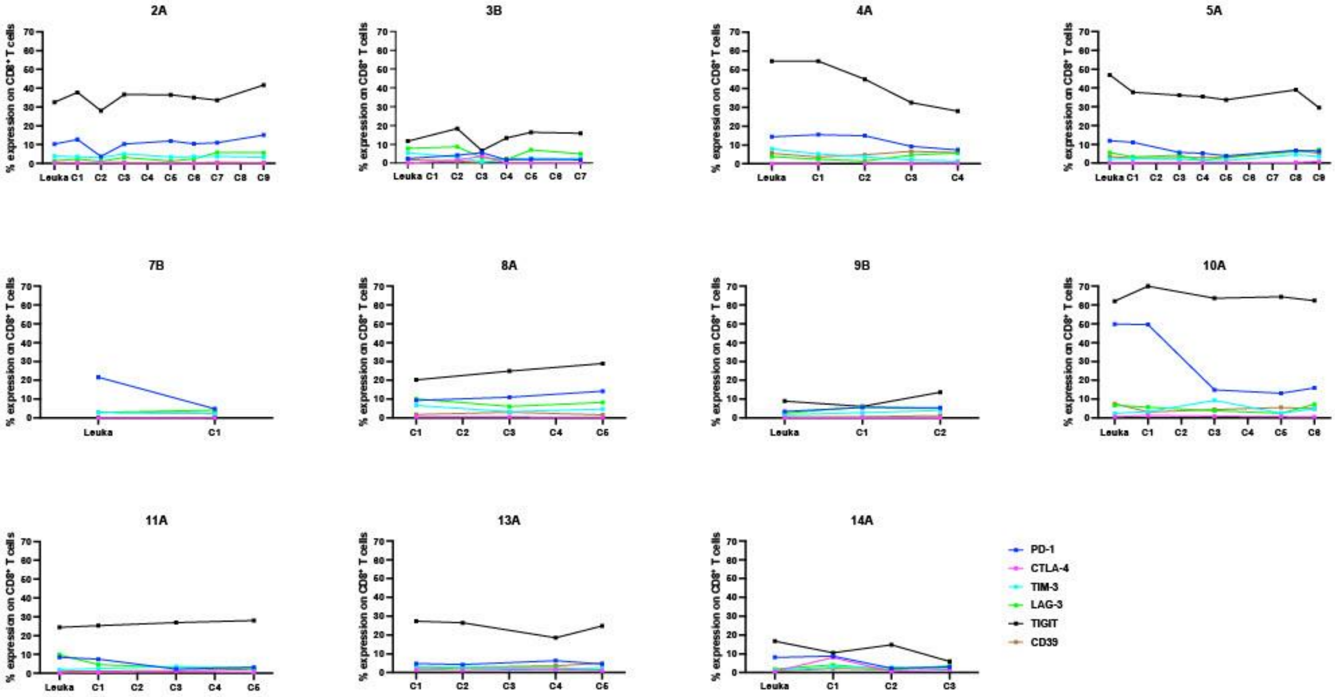

Supplementary Figure 3a: PD-1, CTLA-4, TIM-3, LAG-3, TIGIT, CD39 expression (%) on CD8⁺ T cells over time illustrated per patient. Leuka represents blood analysis at baseline. Blood analysis for every treatment cycle (Q3W) is depicted as C1, C2 etc. A decrease in PD-1 expression in 6 patients (46%) over time is observed, with patient 10A showing a marked decrease from 50% to 13% 12 weeks post-SBRT, while TIGIT expression remained high overall, and LAG3 expression varied.

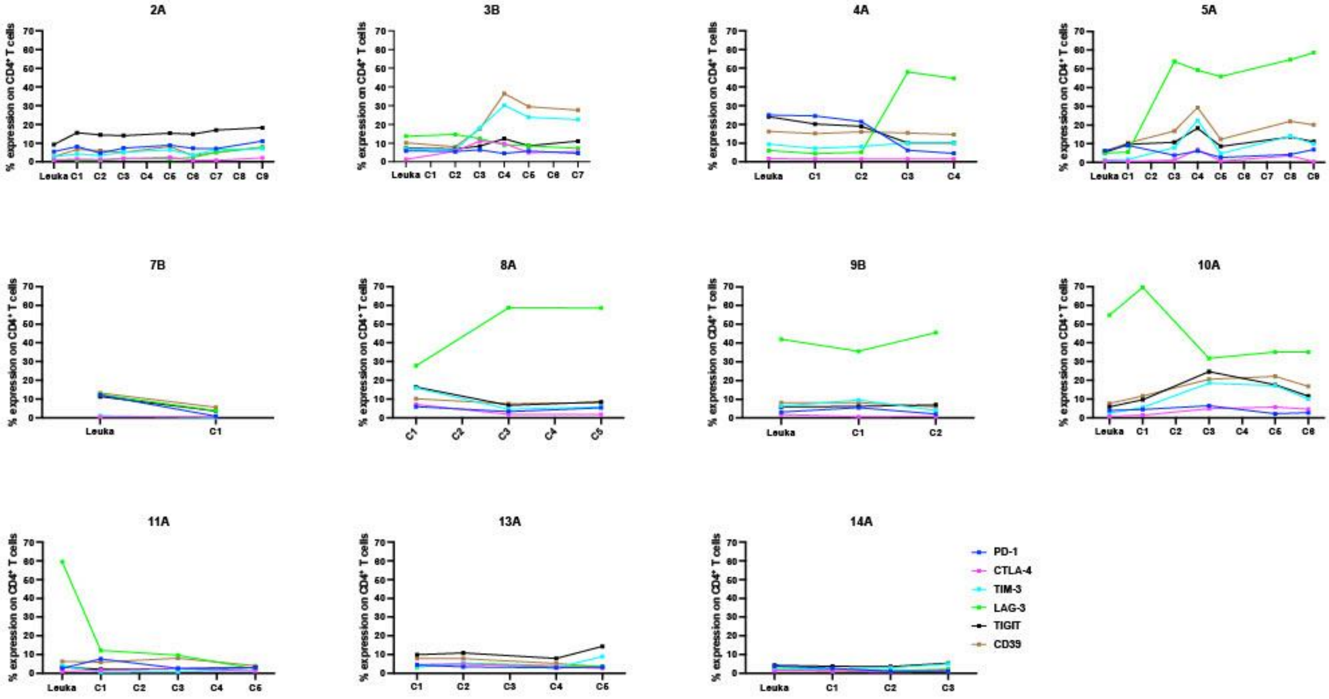

Supplementary figure 3b: PD-1, CTLA-4, TIM-3, LAG-3, TIGIT, CD39 expression (%) on $CD4^+$ T cells over time illustrated per patient. Leuka represents blood analysis at baseline. Blood analysis for every treatment cycle (Q3W) is depicted as C1, C2 etc. LAG3 expression on $CD4^+$ T cells decreased in patient 3A, 7B, 10A and 11A, while an increase was seen in patient 4A, 5A and 8A as compared to baseline. No changes were observed in CTLA-4, TIM3 and CD39 expression.

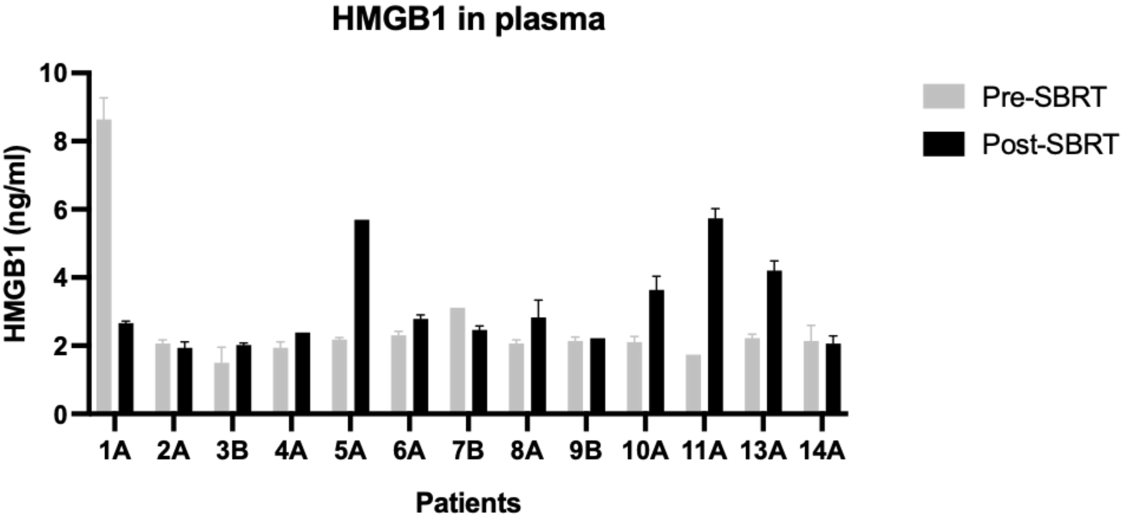

Supplementary Figure 4: HMGB1-levels in plasma in patients pre- and post-SBRT. Depicted as mean with SD. (N=2 (technical replicate)). *Abbreviations: HMGB1: High mobility group box 1 protein, pre-SBRT: HMGB1 previously to stereotactic body radiation therapy, post-SBRT: HMGB1 after stereotactic body radiation therapy. A: arm A (immediate treatment arm), B: arm B (Contemporary control arm).*

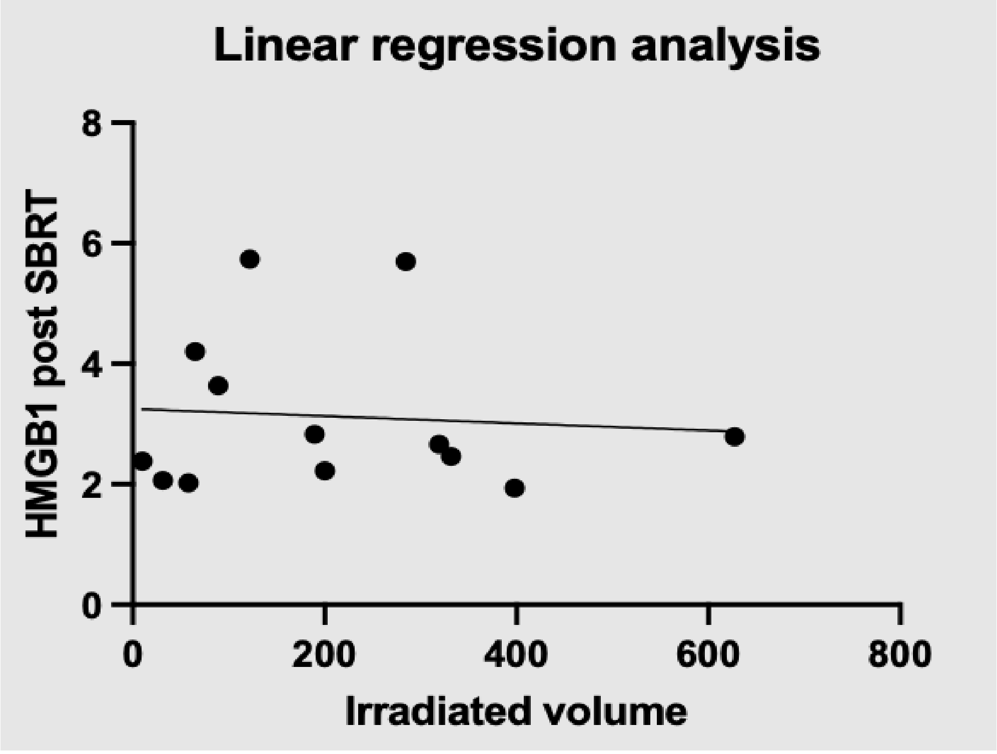

Supplementary figure 5: Linear regression analysis between irradiated volumes of tumor and HMGB1-levels in plasma of patients post-SBRT.

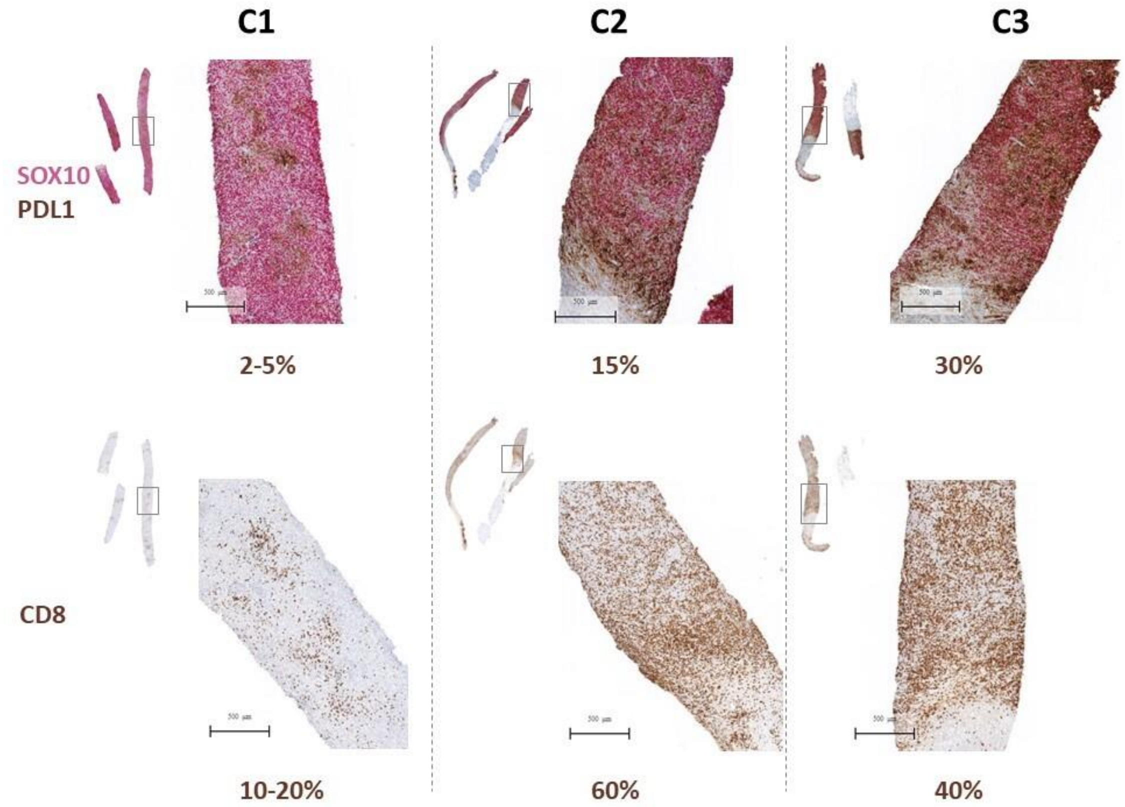

Supplementary figure 6: Immunohistochemistry stainings on tumor biopsies of patient 14A (partial responder), during treatment. Immunohistochemistry staining for SOX10/PDL1(%)- and respectively $CD8^+$ T-cells (%) are shown for every treatment cycle (Q3W), depicted as C1, C2 etc.
