# Supplementary material for: A randomized phase II clinical trial of stereotactic body radiation therapy (SBRT) and systemic pembrolizumab with or without intratumoral avelumab/ipilimumab plus CD1c (BDCA-1)+/CD141 (BDCA-3)+ myeloid dendritic cells in solid tumors
Source: Cancer Immunol Immunother. 2024 Jul 2;73(9):167. doi: 10.1007/s00262-024-03751-0 (PMC11219623; doi:10.1007/s00262-024-03751-0)
Supplement: Supplementary file 2 — Supplementary file2 (DOCX 20 kb) [file 262_2024_3751_MOESM2_ESM.docx]

# Supplementary methods

Cryopreservation of myeloid dendritic cells

After enrichment on the Prodigy device, CD1c (BDCA-1)^+^ and CD141 (BDCA-3)^+^ myDC fractions were concentrated by centrifugation, suspended in a freezing solution (10% DMSO; WAK-Chemie, Steinbach, Germany and 10% human serum albumin; CSL Behring, Mechelen, Belgium), and stored in cryovials at -80°C (container Nalgene® Mr Frosty). Finally the vials were stored in vapor phase of liquid nitrogen. Thawing involved a water bath at 37°C, followed by washing and resuspension in a 4% human serum albumin-0.9% NaCl solution (1-2 ml) before injection.

Determination of lymphocyte subsets and HMGB1 in peripheral blood

Peripheral blood mononuclear cells (PBMCs) were isolated through Histopaque® 1077 (Avantor) density gradient centrifugation according to the manufacturer’s instructions. PBMCs were phenotyped using flow cytometry using the following antibodies: anti-CD3-Viogreen, anti-CD8-Vioblue, anti-CD45RA-VioBright515, anti-CD45RO-APC-Vio770, anti-CD197-APC, anti-CD27-PE-Vio770, CD62L-PE, anti-CD57-APC, anti-CD28-PE, anti-CD25-VioBright515, anti-CD127-PE-Vio770, antiCD279-VioBright515, anti-CD152-APC, anti-CD366-PE-Vio770, anti-CD223-PE, anti-TIGIT-PE-Vio615, anti-CD39-APC-Vio770, Foxp3-APC, Ki67-PE (Miltenyi Biotec), anti-CD4-AF700 (eBioscience), Fixable viability kit Zombie Yellow (Biolegend). Cells were acquired using the BD LSR Fortessa (Beckton Dickinson) and data analysis was performed with Flowjo10.10 (Treestar inc).

HMGB1 levels in the patient’s plasma (kept at -80°C) were determined pre- and post-SBRT using a HMGB1 express ELISA according to manufacturer's instructions (IBL International GmbH).
