# Supplementary material for: A randomized phase II clinical trial of stereotactic body radiation therapy (SBRT) and systemic pembrolizumab with or without intratumoral avelumab/ipilimumab plus CD1c (BDCA-1)+/CD141 (BDCA-3)+ myeloid dendritic cells in solid tumors
Source: Cancer Immunol Immunother. 2024 Jul 2;73(9):167. doi: 10.1007/s00262-024-03751-0 (PMC11219623; doi:10.1007/s00262-024-03751-0)
Supplement: Supplementary file 3 — Supplementary file3 (PDF 873 kb) [file 262_2024_3751_MOESM3_ESM.pdf]

Supplementary Tables

Supplementary table 1: Individual patient tumor type specifications and number of injected myDC for individual patients after thawing and washing.

PATIENT	TUMOR TYPE		NUMBER OF INJECTED MYDC CD1C + CD141 (X 10^6)
**1A**	NSCLC	Stage IV	14,1
**2A**	NSCLC	Stage IV	15,3
**3B**	NSCLC	Stage IV	37,6
**4A**	NSCLC	Stage IV	NA
**5A**	NSCLC	Stage IV	13,6
**6A**	NSCLC	Stage IV	NA
**7B**	NSCLC	Stage IV	6,5
**8A**	Uveal melanoma	Stage IV-M1c	13,2
**9B**	Cutaneous melanoma	Stage IV-M1a	NA
**10A**	Cutaneous melanoma	Stage IV-M1a	23,9
**11A**	Cutaneous melanoma	Stage IV-M1b	17,2
**13A**	NSCLC	Stage IV	15,6
**14A**	Cutaneous melanoma	Stage IV-M1a	23,3
			**Average:** 18.0<br>**Median:** 15,4626

Supplementary table 2: All adverse events

ADVERSE EVENTS n, (%)				
CTCAE v 5.0 grade	**Grade 1 or 2**		**Grade 3**	
	**Arm B**	**Arm A**	**Arm B**	**Arm A**
Abdominal pain		1 (10)		
Adrenal insufficiency				1 (10)
Amenorrhea		1 (10)		
Anemia		1 (10)		
Anorexia	1 (33,3)			
Arthritis	1 (33,3)			
Chills	1 (33,3)			
Conjuctivitis		1 (10)		
Constipation	1 (33,3)			
Cough	2 (66,6)			
COVID		1 (10)		
Diarrhea		1 (10)		
Dyspnea	1 (33,3)	2 (20)		
Eczema		1 (10)		
Fatigue	2 (66,6)	4 (40)		
Fever		1 (10)		
Flu-like symptoms	1 (33,3)			
GERD		1 (10)		
Horner syndrome	1 (33,3)			
Hypertension				1 (10)

Hyperthyroidism		1 (10)		
hypocalcemia		1 (10)		
Hypophysitis				1 (10)
Hypothyroïdism	1 (33,3)			
Infection		1 (10)		
Injection site reaction		3 (30)		
Ischemia cerebrovascular				1 (10)
Lymphopenia		1 (10)		
Malaise		1 (10)		
Muscle weakness lower limb		1 (10)		
Nausea	1 (33,3)	4 (40)		
Paresthesia		1 (10)		
Peripheral sensory neuropathy		1 (10)		
Pleuritic pain		1 (10)		
Pneumonitis	1 (33,3)	2 (20)		1 (10)
Pneumothorax			1 (33,3)	
Pruritus	1 (33,3)			
Nycturie		1 (10)		
Skin infection		1 (10)		
Tumor Pain	1 (33,3)	1 (10)		
Vomiting		1 (10)		
Weight loss		2 (20)		

There were no grade 4 or 5 adverse events
